# N-Terminus Plays a Critical Role for Stabilizing the Filamentous Assembly and the Antifungal Activity of Bg_9562

**DOI:** 10.1128/spectrum.01607-22

**Published:** 2022-08-25

**Authors:** Pankaj Kumar Sahoo, Rahul Kumar, Aruna Rani, Sunil Kumar Yadav, Gopaljee Jha, Deepti Jain

**Affiliations:** a Transcription Regulation Lab, Regional Centre for Biotechnology, NCR Biotech Science Cluster, Faridabad, Haryana, India; b Plant Microbe Interactions Laboratory, National Institute of Plant Genome Research, New Delhi, India; University of Guelph

**Keywords:** Bg_9562, tail assembly chaperone, spiral fibers, antifungal, mycophagy

## Abstract

Bg_9562, a prophage tail-like protein was earlier shown to be required for bacterial mycophagy by Burkholderia gladioli strain NGJ1. The purified protein exhibited broad-spectrum antifungal activity; however, the structural and mechanistic details vis-à-vis its activity remained elusive. In this study, we have structurally characterized the protein Bg_9562 using negatively stained transmission electron microscopy, molecular modeling and mutagenesis. We find that Bg_9562 shows structural similarity to Gp13, a tail assembly chaperone. The transmission electron microscopy revealed that, Bg_9562 forms long flexible tubular structures. Molecular modeling of the filament like structure divulges that the inter subunit contacts are meditated largely through hydrophobic interactions. Using mutagenesis, we demonstrate that the N-terminal residues of the protein when deleted results in reduced activity and destabilization of filament formation. Overall, structure-function analysis opens up avenues for further utilization of the protein as a potent antifungal molecule.

**IMPORTANCE** Burkholderia gladioli strain NGJ1, isolated from healthy rice seedling, was earlier demonstrated to have mycophagous properties on a broad range of fungi, including Rhizoctonia solani, a causal agent of deadly sheath blight disease of rice. The purified Bg_9562 protein exerts broad-spectrum antifungal activity. The protein also inhibits the growth of laboratory strain of Candida, an opportunistic human pathogen. In this study, we structurally characterize Bg_9562 using a combination of negative staining transmission electron microscopy, molecular modeling, mutagenesis, and functional antifungal assay. We show that the protein assembles into long filament like structures stabilized by N-terminus residues and this region is important for its activity. Our study has implications in utilizing Bg_9562 or its derivatives as antifungal molecule(s) which will provide environmentally friendly control of fungal diseases of plants and animals.

## INTRODUCTION

Bacteria coexist with fungi in complex polymicrobial ecosystems, including soil, rhizosphere, and microbiota of plants and humans. They interact with each other either synergistically or antagonistically. Mycophagy is one such interaction, wherein some of the bacterial species have ability to forage over fungi and utilize fungal biomass as nutrient source (1). This facilitates the bacteria to compete for the limited resources in the hostile environment and occupy particular niche in the host. The examples of fungivorous bacteria include species of Pseudomonas, *Collimonas,* and *Burkholderia* (2). They often utilize type III secretion system (T3SS) machinery to deliver the effector proteins into neighboring cells to get selection advantage during inter microbial competition (3, 4). These effector proteins presumably disrupt the fungal cells (cell wall as well as cell membrane) and enable the bacteria to utilize fungal biomass as nutrient source.

Burkholderia gladioli strain NGJ1 that was isolated from a healthy rice seedling, demonstrated mycophagy on a broad range of fungi, including Rhizoctonia solani, a causal agent of deadly sheath blight disease of rice (5). The NGJ1 utilizes T3SS to potentially deliver a cocktail of antifungal effector proteins into fungal cells to kill them (4, 5). Notably, a prophage-tail like protein (Bg_9562) has been shown to be a bonafide T3SS effector and is required for mycophagous ability of NGJ1 on R. solani (5). The purified Bg_9562 protein exerts broad-spectrum antifungal activity on not only R. solani, but also on various other fungi (generally having chitin rich cell wall), including an oomycetes pathogen, *Phytophthora* sp (having nonchitinous cell wall). The protein also inhibits the growth of laboratory strain of *Candida*, an opportunistic human pathogen. Considering the broad range of antifungal activity on plant and animal pathogenic fungi, it has been emphasized that Bg_9562 can be developed as a novel antifungal molecule. However, the structural and mechanistic details of antifungal activity of Bg_9562 remain elusive and are required to further establish Bg_9562 as novel antifungal molecule.

It is to be highlighted that fungal diseases of plants as well as animals are posing greater challenge for mankind (6). Due to excessive antifungal usages, many of the fungal pathogens have evolved tolerance against commonly used antifungal drugs/chemicals and rendered them noneffective (7). This warrants development of novel antifungal molecules, and many of the antifungal peptides are being identified from microbial/plant sources. Understanding the structure-function relationship has been helpful in establishing mechanism responsible for antifungal activity (8, 9). These studies have shown that antifungal peptides are often amphipathic, Cys rich molecules, carrying an overall positive charge and have stable scaffolds. They function through a variety of different mechanisms, including cell wall binding, membrane permeabilization, interaction with intracellular targets and induction of signaling cascades (10).

In this study, we structurally characterize the antifungal protein Bg_9562 using a combination of molecular modeling, negative staining transmission electron microscopy, mutagenesis, and functional antifungal assay. The protein assembles into long filament like structure that is largely stabilized through inter-subunit hydrophophic interactions. Additionally, we demonstrate that the N-terminal amino acids residues of the Bg_9562 protein are important for antifungal activity and might be involved in further stabilization of the long tubular structure. The structural model is further validated through various deletion constructs of the protein.

## RESULTS

### Prediction of Bg_9562 structure with AlphaFold2.

**FIG 1:**
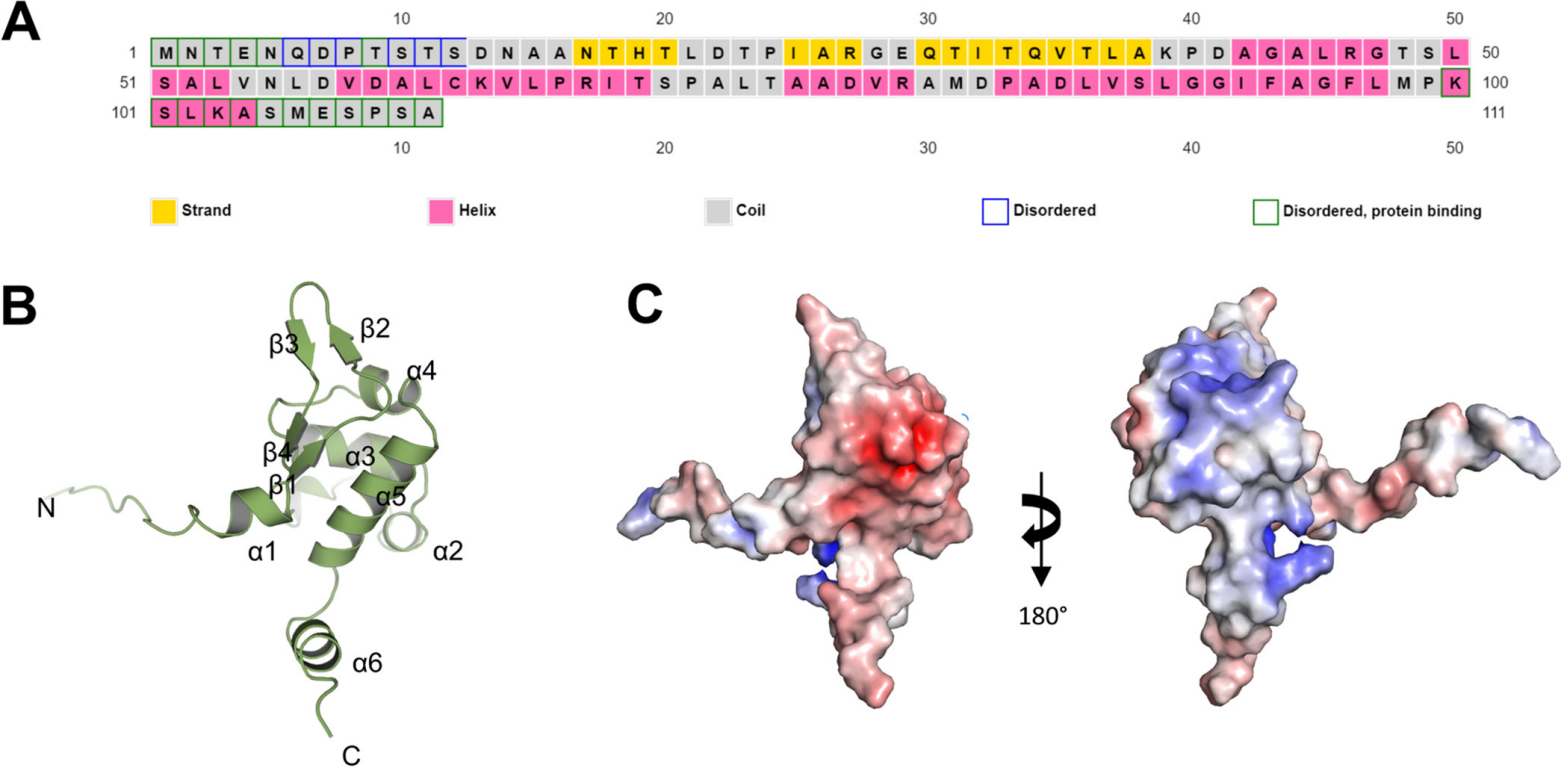
Secondary structure prediction and 3D model of Bg_9562. (A) Secondary structure of Bg_9562 was predicted using the PSIPRED server (http://bioinf.cs.ucl.ac.uk/psipred/). (B) Ribbon diagram of predicted AlphaFold2 model of Bg_9562 is shown and the secondary structural elements are labeled. (C) Electrostatic surface potential of Bg_9562 is displayed.

A BLAST search reveals that Bg_9562 (111 amino acids), does not show any significant sequence homology to proteins whose structures have been determined and deposited in the protein data bank (PDB) but places it in the phage tail assembly chaperon superfamily. Structure prediction using PSIPRED (11) revealed intrinsically disordered regions at the N and the C terminus of the protein with largely helical content of 43%, a low β-strand content of 14% and 43% of coils (Fig. 1A). We then analyzed the three-dimensional structure of Bg_9562 predicted with AlphaFold2 (12). AlphFold2 predicts the structure based on preinstalled databases and creates five predicted models (13, 14). The model that shows highest per-residue confidence score is determined as the final structure. The three-dimensional model of Bg_9562 monomer comprises of a single domain with the core consisting of six alpha helices and four beta strands (Fig. 1B). The N-terminal residues 1 to 12 are disordered while the core of the protein is hydrophobic. Surface electrostatic potential indicates a negatively charged surface on one side of the molecule and a positively charged surface on the reverse (Fig. 1C).

### The structure of Bg_9562 resembles tail assembly chaperone proteins of phages.

**FIG 2:**
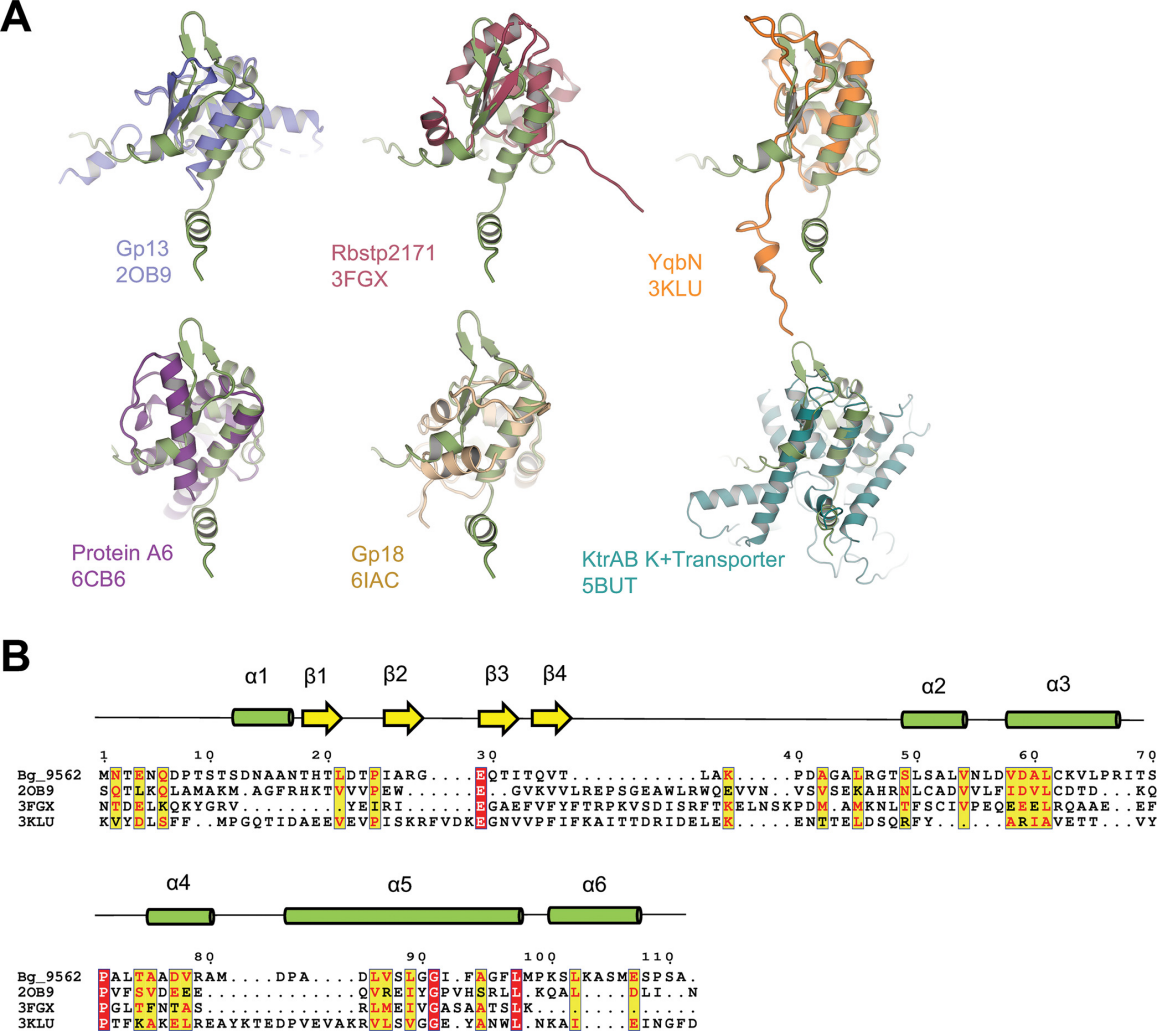
3D-structure based homologues of Bg_9562 by DALI. (A) Structural superimposition of top hit proteins with Bg_9562 (green) obtained with DALI are shown. (B) Sequence alignment of Bg_9562 is shown for the structural homologs hits obtained by DALI and displaying more than 10% sequence identity. The residues in red are similar and those enclosed in red box indicate absolute conservation. The secondary structural elements are shown on top of the sequences the basis of model generated by AlphaFold2 for Bg_9562, green cylinders represent alpha helix and yellow arrows represent beta sheet.

With the aim to identify closely related structures, a Distance Matrix Alignment (DALI) search using the AlphaFold2 predicted structure of full-length Bg_9562 yielded few hits (15). The top 6 proteins structures with the DALI Z-scores ranging from 4.5 to 2.8 are presented in Table 1. The Z-score is calculated as a sum of the equivalent residue-wise intermolecular distances between two proteins. The search disclosed that Bg_9562 shares structural similarity to the members of tail assembly chaperones (TAC). The highest Z-score was of Gp13, a TAC protein of phage HK97 (PDB: 2OB9) with the DALI score of 4.5 (16). The root mean square deviation (rmsd) of superimposition of the two proteins was 4 Å over 79 Cα positions (Fig. 2A). YqbN (PDB: 3KLU) from Bacillus subtilis and Rbstp2171 (PDB: 3FGX) from Geobacillus stearothermophilus both show Z-score of 4.4 and rmsd of 5.2 Å and 4.9 Å, respectively, with Bg_9562 (Fig. 2A). All the above three proteins are phage tail assembly chaperones and share the structural protein core with Bg_9562. The next best structural match was with the N terminus of the viral membrane assembly protein A6 (PDB: 6CB6) with Z-score of 3.7. This is followed by Gp18 protein of bacteriophage P68 (PDB: 6IAC) and KtrAB K^+^ transporter protein from B. subtilis (PDB: 5BUT) both of which showed a low Z-score of 2.8 as only a part of the protein shows structural similarity with Bg_9562 (17–19).

**TABLE 1:** Top matches from DALI search of Bg_9562 Alphafold2 model

Proteins	Function	PDB iD	Z-score	RMSD (Å)	Similarity (%)	Identity (%)
gp13	Tail assembly chaperone	2OB9	4.5	4	28	19
Rbstp2171	Uncharacterized protein	3FGX	4.4	4.9	26	15
YqbN	Uncharacterized protein	3KLU	4.4	5.2	30	15
N terminus of protein A6	Viral membrane assembly protein	6CB6	3.7	3.7	19	10
gp18	Portal and tail of native bacteriophage P68	6IAC	2.8	4.4	14	6
KtrAB K+ transporter	Potassium uptake protein	5BUT	2.8	4.8	12	6

Bg_9562 displays high sequence divergence with the proteins identified through DALI. The sequence identity among these proteins is limited and ranges from 19% to 6% as calculated in ClustalW (20). We performed a multiple sequence alignment of Bg_9562 with the hits obtained from DALI. Out of the 6 proteins, the Bg_9562 shows highest sequence identity of 19% (28% similarity) with the Gp13 of HK97 phage (PDB ID: 2OB9) (Fig. 2B). Protein A6, Gp18 and the KtrAB were excluded from the alignment as they display less than 10% sequence identity (Table 1).

### Bg_9562 forms higher order oligomers.

**FIG 3:**
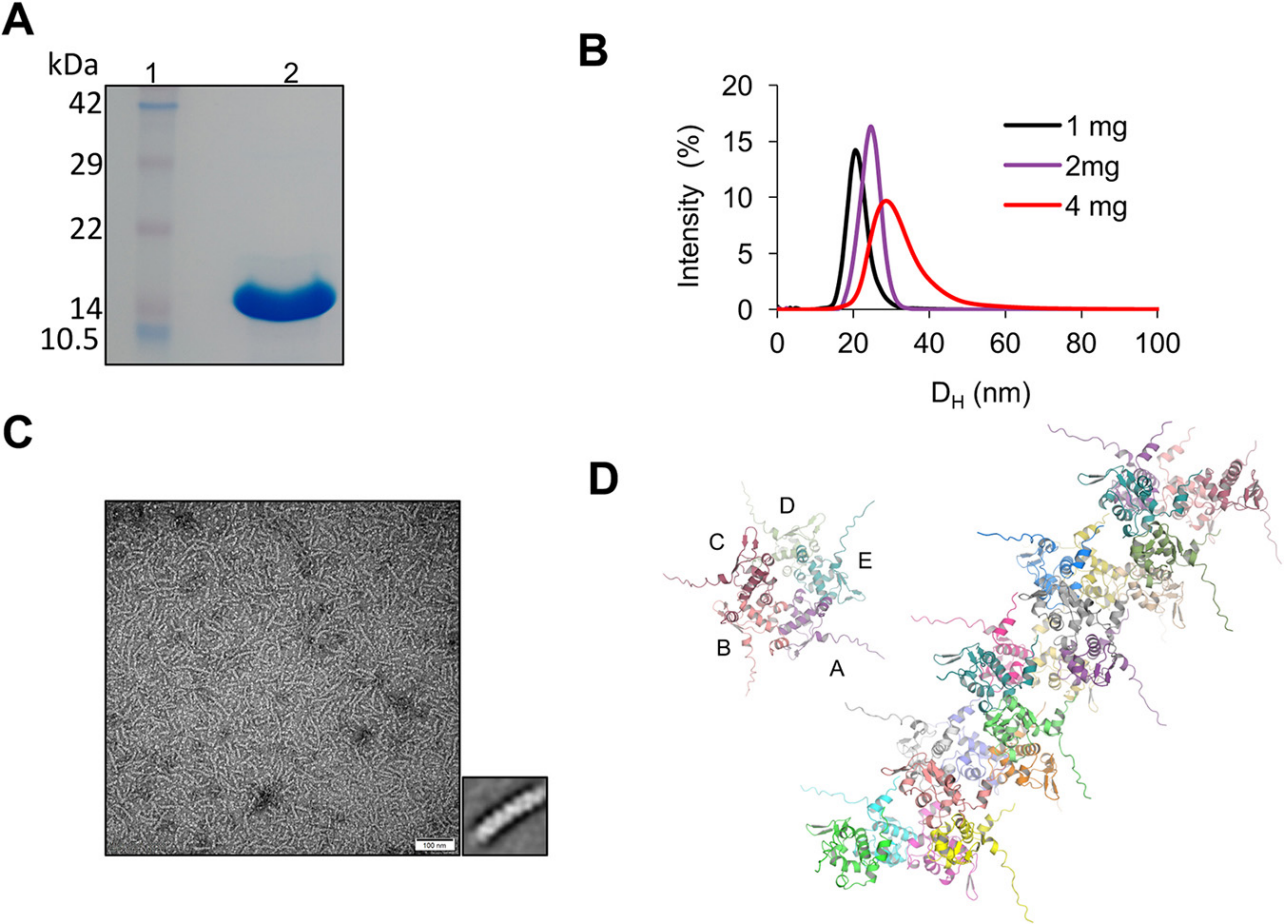
Structure of the oligomeric assembly. (A) SDS-PAGE gel showing purified wild-type Bg_9562. (B) Distribution of apparent hydrodynamic diameter of Bg_9562 at 1 mg/mL (black), 2 mg/mL (violet) and 4 mg/mL (red) obtained by DLS are shown. (C) Representative raw electron micrograph of negatively stained of Bg_9562 (left side) with 100 nm scale bar. Small inset shows representative 2D class average of the individual fiber like structure of the Bg_9562. (D) Top view of the fibrous structure showing arrangement of five subunits labeled from A–E and structural Alphafold2 model showing the possible arrangement of each monomer in the fibrous structure.

To further gain structural insights into the function of Bg_9562, we expressed and purified the full-length protein. The SDS-PAGE gel of the purified protein is shown in Fig. 3A. Despite extensive screening, for crystallization of the full-length protein; crystals were not obtained. We further employed dynamic light scattering (DLS) to assess the oligomerization state of Bg_9562 as a function of protein concentration. DLS measures the apparent hydrodynamic diameter (D_H_) of the protein. At 1 mg/mL, the protein showed a single peak with the D_H_ value of 19 nm. We observed that the D_H_ values of full-length Bg_9562 as measured by DLS increases with increase in the protein concentration (Fig. 3B). The D_H_ increases to 23.5 nm and 27 nm at 2 mg/mL and 4 mg/mL, respectively (Fig. 3B). This indicates that the protein forms higher order oligomers in concentration dependent fashion.

### Bg_9562 assembles into long spiral fibers.

To investigate the assembly formed by Bg_9562, purified Bg_9562 was analyzed by negative stain transmission electron microscopy (TEM). The raw negatively stained electron micrographs show that the protein self assembles into long, flexible filaments which varied in length. Averaged side view (Fig. 3C, inset) shows that these filaments had ordered helical morphology. The bent fibers suggest flexibility in the tubular filaments. Since the crystal structure of the protein was unavailable, to further probe the oligomeric state of Bg_9562, we predicted the quaternary assembly of the Bg_9562 using AlphaFold2. Bg_9562 assembled into spirals such that each turn of the spiral was composed of 5 subunits (Fig. 3D). The internal diameter of the spiral assembly was roughly 25 Å. The self-assembly of the protein is facilitated largely through inter-subunit hydrophobic interactions. Interaction interface involves the three loops from one subunit—one between α4 and α5, the second between α2 and α3, and the third between β strand 1 and 2. These form the interface with the loops between α5 and α6 and between β strand 4 and α2 of the adjacent subunit (Fig. 3D). Overall, there was good correspondence between the TEM image of Bg_9562 and the structural model of the fibrous filaments predicted through AlphFold2.

### Over expression and purification of Bg_9562 truncated constructs.

**FIG 4:**
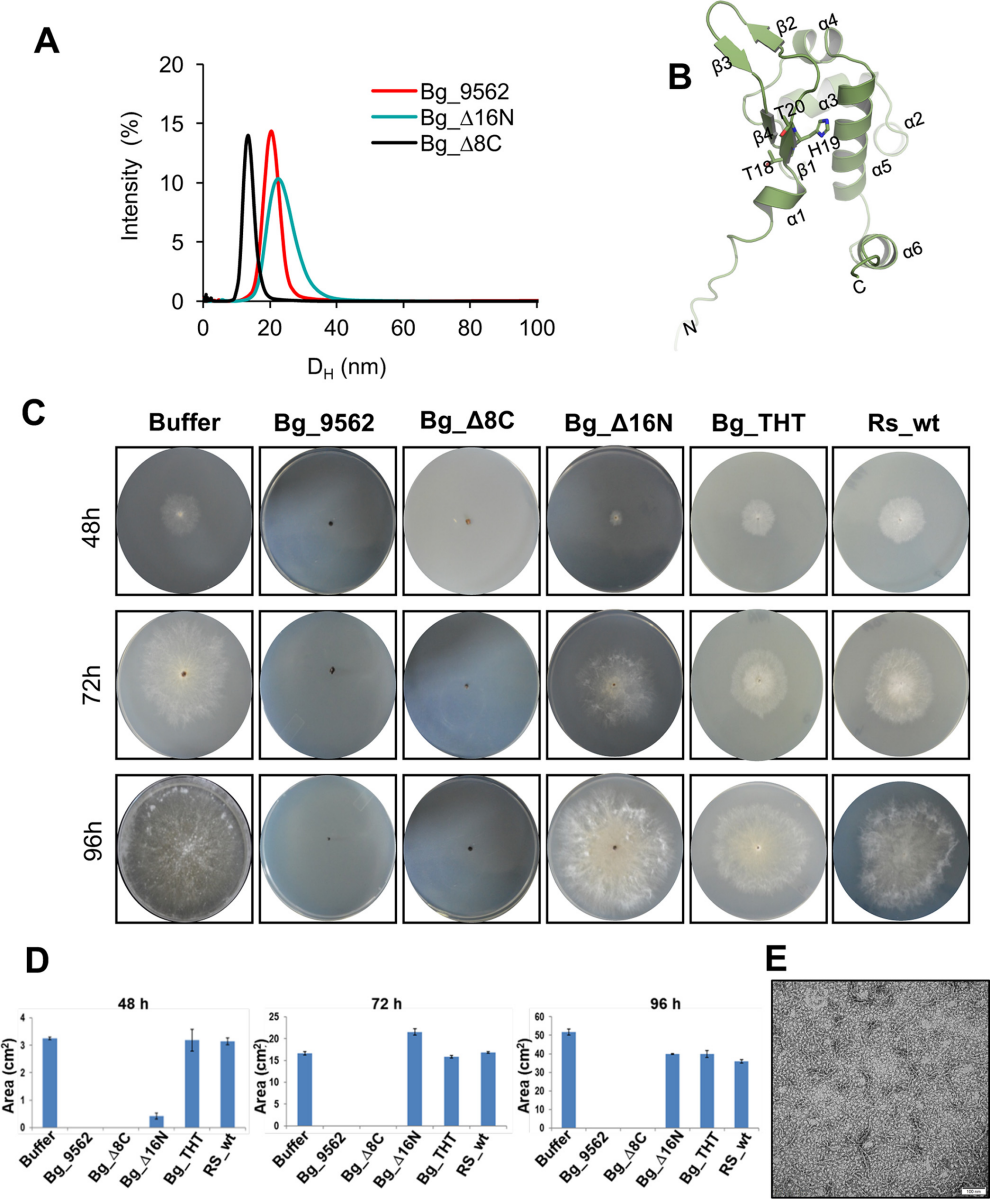
Activity profile of various mutants. (A) Hydrodynamic diameter (X-axis) of Bg_9562 (red line), Bg_Δ16N (blue line) and Bg_Δ8C (black line) plotted as a function of intensity. (B) THT motif in Bg_9562 is shown in stick representation. (C) Effect of wild-type Bg_9562 and wild-type homolog from *Ralstonia solanacerum* (Rs_wt) protein and bg_9562 variants on growth of R. solani on fresh PDA plates at 48 h, 72 h and 96 h time points. (D) R. solani growth measured in terms of mycelial growth area (cm^2^) on PDA plates, at different time points. Data represents mean and standard error of three independent biological replicates, each having three technical replicates. (E) Representative raw electron micrograph of negatively stained of Bg_Δ16N with 100 nm scale bar.

While the model indicates that the N-terminus and C-terminus region are not in contact with the adjacent subunits, we further probed the role of these regions in structure and antifungal activity. Thus, the N and C-terminal truncated constructs of Bg_9562 were designed, cloned, expressed in E. coli and purified. These constructs were designed on the basis of structure prediction of Bg_9562. The structure prediction shows 16 residues from the N terminus and about 7 to 8 residues from the C terminus of the protein were disordered (Fig. 1A). Thus, we designed Bg_Δ8C and Bg_Δ16N where 8 amino acids from the C terminus or 16 amino acids from the N terminus were deleted, respectively. The deletion constructs were purified using the same protocol employed for wild-type protein. To assess the impact of the amino acids deletions from the N and C-terminal on the size of the protein, DLS was performed on the truncated proteins. The investigation shows that there was reduction in size of the construct that was truncated from the C terminus (Fig. 4A). On the other hand, truncation from the N terminus results in an increase in the distribution width and decrease in the peak intensity indicating larger size particles and nonhomogeneous mixture in the protein sample, respectively.

### Antifungal activity of Bg_9562 constructs.

We evaluated the antifungal activity of the purified wild-type Bg_9562 and the N- and C-terminus truncated variants on R. solani using a functional assay. It was observed that the treatment with wild-type Bg_9562 and the Bg_Δ8C prevented the fungal growth on potato dextrose agar (PDA) plates. On the other hand, treatment with Bg_Δ16N variant led to partial growth inhibition of R. solani during 48h of incubation. Moreover, upon further incubation (during 72 h and 96 h) growth of the treated R. solani was comparable to that of buffer-treated ones (Fig. 4C and D). This emphasized that Bg_Δ16N is compromised in antifungal activity. This also suggested that the N terminus of the protein is essential for the activity whereas truncations of C terminus have no effect on the antifungal activity of Bg_9562. Previously we have reported that N-terminal amino acids of Bg_9562 homologs in different bacteria exhibit significant diversity (Yadav et al. 2020). Sequence alignment of Bg_9562 homologs reflected that the “THT” motif at 18 to 20 amino acid is conserved in the Bg_9562 homologs of different Burkholderia gladioli strains but it varies in that of other plant associated bacteria (Ralstonia solanacearum and Stenotrophomonas maltophilia) (Fig. S1). We observed that this motif is present on the first beta strand in the protein (Fig. 4B). To test its importance, we designed a variant, Bg_THT protein, where the motif “THT” was substituted with “AVA.” Evidently these substitutions resulted in loss of antifungal activity, as the growth of Bg_THT treated R. solani was comparable to that of buffer-treated ones, at each of the time points (Fig. 4C and 4D).

Further, we tested the ability of the *R. solanacerum* homolog of Bg_9562 (Rs_wt) for antifungal activity. To our surprise the protein did not exhibit antifungal activity on R. solani (Fig. 4B). Analysis of the Bg_9562 sequence and its *Ralstonia* homolog (Rs_wt), reflected that the protein from *R. solanacerum* was 11 amino acids shorter from the N terminus compared to the one from *B. gladioli* (Fig. S2). Additionally, the “THT” sequence is “TVT” in *R. solanacerum*. This further reinforces that the N-terminal residues of Bg_9562 are important for antifungal activity.

### Deletion of N terminus destabilizes the filaments.

Next we asked if the N-terminus residues were important in stabilizing the filamentous assembly of the protein. Negatively stained sample of the Bg_Δ16N was examined by transmission electron microscopy. The electron micrograph shows that although, this variant was able to form seemingly identical filaments to the ones formed by full-length protein, but they were fewer in number (Fig. 4E). Additionally, the deleted variant leads to the formation of heterogeneous species with significantly large population forming rings. This observation is consistent with the DLS of this variant and confirms that the N terminus might stabilize the filament formation (Fig. 4A).

## DISCUSSION

A rice associated Burkholderia gladioli strain NGJ1 has been shown to deploy one of the effector protein Bg_9562 using type III secretion system (T3SS) to forage over fungi and gets an added advantage to proliferate under hostile and competitive environment (21). The Bg_9562 protein demonstrates potent and broad-spectrum antifungal activity; although it does not contain any toxic or lytic domain. The protein is encoded in a cryptic bacteriophage encoding gene cluster wherein head assembly proteins are absent (5). Although, the protein demonstrates weak sequence similarity with prophage tail-like proteins; however, in the absence of structural details, it was intriguing how a prophage tail-like protein exerts antifungal activity.

We observed that the purified Bg_9562 protein self assembles into long filamentous flexible fibers. Using a combined modeling, microscopy, and mutagenesis approach we have proposed a three-dimensional structural model for the Bg_9562 which assembles into long conduit spiral structures, formed through hydrophobic interactions. The N terminus of the Bg_9562 is intrinsically disordered and rich in polar amino acids. The intrinsically disordered regions are widespread in proteins and play crucial roles in molecular recognition, assembly and stabilization of complexes such as in case of neurofilaments or large multiprotein complexes such as elastin (22, 23). They have been known to wrap around binding partners and contribute significantly to binding affinity and specificity (24, 25). Interestingly deletion or substitution of residues present at the N terminus led to decrease in the antifungal activity of Bg_9562, providing compelling experimental evidence for the role of N terminus in antifungal activity. Further, TEM indicates that the N terminus might be relevant for stabilization of the spiral assembly of the protein.

Structural characterization of Bg_9562 also unveiled substantial resemblance with tail assembly chaperones (TAC) proteins of bacteriophages even in the absence of significant sequence similarity. The TAC proteins function as molecular chaperons and assist in assembly of the tails in the long-tailed phages (26, 27). The multicomponent long tails of the bacteriophage are either contractile or noncontractile (27, 28). The assembly of tails begins with the formation of tail tip. The tape measure proteins are essential determinants of the length of the phage tails and their polymerization requires a scaffold formed by the chaperone proteins. Generally, the tail assembly chaperones comprise of two open reading frames (ORFs) resulting in production of two proteins one of which is produced 3 to 4% of the other (16). The fixed ratio of the proteins produced as a result of frameshift mechanism is essential for efficient assembly of the phage tail (26). It remains to be explored whether during mycophagous interaction, the Bg_9562 is produced from two different ORFs.

Analysis of the quaternary assembly of the TAC family of proteins disclosed that they all oligomerize into long tubular structures with a central pore (16). The Gp13 of HK97 forms octameric rings utilizing head to tail arrangement of monomers such that the N terminus of one monomer docks into the adjacent subunit (16). The inter-subunit interactions in the YqbN are also formed through the N terminus of the protein forming spirals in crystal lattice. The Rbstp2171 forms dimers utilizing the C-terminus helix. The dimers then pack back-to-back forming a spiral. Although all the TAC family of protein form tube like structures, the inter-subunit interactions are unique in different proteins. The oligomeric assembly is facilitated by either the N or the C terminus of the protein. The structure of Gp18 of the phage 68 is mushroom shaped and forms a channel made from the dodecamer and is unlikely to form spiral. The KtrB from the KtrAB complex forms octameric rings. However, the viral membrane assembly protein A6 does not oligomerize.

We postulate that N terminus of Bg_9562 is crucial for antifungal activity. The antiparallel beta strand appears to be relevant as the mutations in this region were detrimental for the activity. The motif “THT” is present in the first beta strand which is surface exposed and its mutation abrogated the antifungal activity. Although, the N-terminus deletion destabilized the filaments, mutation of THT motif is less likely to weaken the spiral arrangement. It is therefore speculated that the spiral organization of the protein might play a role in improving recognition by the fungal membrane. The fibers behave as multivalent ligands which possibly leads to an increase the overall functional affinity toward the fungal membrane.

Considering the broad-spectrum antifungal activity of Bg_9562, we emphasize that the protein can be effectively deployed to control fungal diseases of plants and animals. Moreover, it can also be deployed as a transgene to impart fungal disease tolerance in crop plants, particularly, against R. solani infection. It is to be noted that R. solani is a deadly fungal pathogen of many agriculturally important plant species, including rice, tomato, potato and maize (29). None of the plant species are known to harbor practical level of disease resistance and hence effective disease control remains challenging (30). In rice, R. solani causes sheath blight disease which accounts for up to 50% yield loss under favorable environmental conditions and the disease is predominantly managed with use of agrochemicals (fungicides), many of which have pleotropic adverse effect on the heath of the consumers as well as the soil (31). Our study emphasizes that Bg_9562 or its derivatives can be further explored as antifungal molecule(s) for effective, environmentally friendly and sustainable control of sheath blight disease.

## MATERIALS AND METHODS

### Cloning and protein purification.

The wild-type (WT) Bg_9562 was cloned into pET28a vector as reported earlier (5). The Bg_Δ16N and Bg_Δ8C constructs were cloned into modified pET28b vector with N-terminus His tag. The primers used are given in Table S1. The gene sequence of Bg_THT wherein the nucleotide sequences corresponding to the “THT” motif at 18 to 20 amino acid of wild-type Bg_9562 was substituted with “AVA” encoding nucleotides and the Ralstonia solanacearum homolog of Bg_9562 (Rs_wt; WP_071615213) were synthesized and subcloned into pET28a expression vector using NdeI-HindIII restriction sites by the service provider (General Biosystems, Inc. USA).

The wild-type Bg_9562, its variants (Bg_ Δ16N, Bg_ Δ8C, Bg_THT), and Rs_wt were all expressed in E. coli BL21 (DE3) cells. The cells were grown in 3 L, LB medium supplemented with appropriate antibiotics and incubated at 37°C with shaking at 180 rpm to mid exponential phase until the OD_600_ reached 0.7 to 0.8. The culture was induced with 1 mM IPTG and incubated at 37°C for 4 h. The culture was harvested by centrifugation at 6000 rpm for 30 min. The cell pellet was resuspended in lysis buffer. The cells were lysed by sonication (Sonics, vibra cells) and the lysate was harvested by centrifugation at 12000 rpm for 60 min. The filtered lysate was loaded onto a charged 5 mL HiTrap Ni-NTA column (GE Healthcare) connected to an ÄKTA purifier system (GE Healthcare) equilibrated with Buffer A (25 mM Tris pH 8, 500 mM NaCl, 5% glycerol, 2 mM β-mercaptoethanol, and 10 mM Imidazole). The bound protein was eluted by using step gradient of buffer B (25 mM Tris pH 8, 500 mM NaCl, 5% glycerol, 2 mM β-mercaptoethanol and 1 M Imidazole). The affinity purified Bg_WT, Bg_Δ16N and Bg_Δ8 were further purified by gel-filtration chromatography using a S300 column equilibrated with gel filtration buffer (25 mM Tris pH 8, 500 mM NaCl, 5% Glycerol, 2 mM DTT, 100 mM Imidazole) in ÄKTA fast protein liquid chromatography system. The elution profile was monitored with absorbance at 280 nm and 214 nm. The fractions were checked on the gel and were snap-frozen using liquid nitrogen and stored at −80°C.

### Dynamic light scattering.

The size distribution analysis experiment was performed by using Malvern Zetasizer Nano ZS instrument (Malvern) at 14°C. For Bg_WT a range of concentration from 1, 2 and 4 mg/mL was used to evaluate the variation in particle size. Bg_Δ8C, Bg_Δ16N variants were used at 1 mg/mL in the buffer containing 25 mM Tris (pH 8), 500 mM NaCl, 5% glycerol, 300 mM imidazole. Correlation data obtained with Zetasizer software (Malvern) were plotted in Excel.

### Transmission electron microscopy.

The Bg_WT and Bg_Δ16N protein was diluted to 0.4 mg/mL in the buffer (25 mM Tris-HCl pH 8, 500 mM NaCl, 5% glycerol, 2 mM β-mercaptoethanol) for negative staining. Briefly, a carbon-coated copper grid was glow discharged for 50 s, 4 μL of protein sample was added to the grid and kept for 1 min. The excess sample was blot dried using Whatman filter paper. Grid was washed with water (30 μL), thrice for 4 sec. each and blot dried. Two drops (20 μL) of 2% uranyl acetate solution was added to a parafilm. Staining was performed for 5 sec. in first drop of stain and then for 45 sec. in second drop. Excess staining solution was blot dried and grids were analyzed under a transmission electron microscope.

### *In silico* analysis of Bg_9562.

Secondary structure of Bg_9562 was predicted using PSIPRED server (11). Both monomer as well as fibrous model of Bg_9562 was generated using AlphaFold2 (12, 14). Bg_9562 model was used to search for structural homologues in the PDB database using DALI server (15). Each homologous structure was superimposed on to Bg_9562 using UCSF CHIMERA software (32). Electrostatic surface potential of monomer and oligomer of Bg_9562 was generated by using APBS plugin of PyMol 2.3 software (https://pymol.org/). Protein sequences were aligned using the T-Coffee server. The aligned sequences were further graphically enhanced using ESPript 3.0 server. The figures were generated using PyMol 2.3.

### Antifungal assay.

Antifungal activity of Bg_9562, Bg_Δ8C Bg_Δ16N, Bg_THT and Rs_wt was assayed on Rhizoctonia solani strain BRS1, as described earlier (5). Briefly, the R. solani sclerotia were treated with 15 μg/mL of each of the purified protein. Buffer treated sclerotia (Phosphate buffer saline, pH 7.4; 10 mM) were used as control. Upon treatment, the sclerotia were placed on PDA (Himedia, India) plates and incubated at 28°C for further growth. The mycelia growth was monitored at different time intervals (24h, 48h and 72h) and area of the mycelial lawn was estimated. A minimum of five sclerotia were analyzed per treatment and each experiment was independently repeated at least three times.
