# Supplementary material for: N-Terminus Plays a Critical Role for Stabilizing the Filamentous Assembly and the Antifungal Activity of Bg_9562
Source: Microbiol Spectr. 2022 Aug 25;10(5):e01607-22. doi: 10.1128/spectrum.01607-22 (PMC9603447; doi:10.1128/spectrum.01607-22)
Supplement: Supplemental file 1 — Fig. S1 and S2 and Table S1. Download spectrum.01607-22-s0001.pdf, PDF file, 1.4 MB [file spectrum.01607-22-s0001.pdf]

Figure S1

```
                                  16 aa         18-20 aa
WP_124093616.1_B.gladioli         MNTENQDPTSTSDNAENTHTLDTPIARGEQTITQVTLTKPDAGALRGTSLSALVNLDVDA        60
WP_060004506.1_B.gladioli         MNTENQDPTSTSDNAANTHTLDTPIARGEQTITQVTLAKPDAGALRGTSLSALVNLDVDA        60
WP_047836209.1_B.gladioli_NGJ1    MNTENQDPTSTSDNAANTHTLDTPIARGEQTITQVTLAKPDAGALRGTSLSALVNLDVDA        60
WP_047836209.1_B.gladioli         MNTENQDPTSTSDNAANTHTLDTPIARGEQTITQVTLAKPDAGALRGTSLSALVNLDVDA        60
WP_013696218.1_B.gladioli_BSR3    MNTENQDPTRTSDNAANTHTLDTPIARGEQTITQVTLAKPDAGALRGTSLSALVNLDVDA        60
WP_126928254.1_S.maltophilia      -----MTAKTNTAIAPNTITLDYPIQRGEQTIETITLRKPNAGELRGIKLVDLLQMDVGA        55
WP_071615213.1_Rs_wt              -----------MDNQTVTVTLDTPIKRGEQAITELTLRKPGAGELRGVSLMDLMRMEVAA        49
WP_197369877.1_R.solanacearum     -MPQLFIALTTMEKKTATITLDTPITRGEQTISTITVRKPGAGELRGVSLMDLMRMDVTA        59
                                                   * *** ** ****:*  :*: **.** *** .*  *:.::* *

                                                                                 8 aa
WP_124093616.1_B.gladioli         LCKVLPRITSPALTAADVRAMDPADLVSLGGIFAGFLMPKSLKASMESPSA-------- 111
WP_060004506.1_B.gladioli         LCKVLPRITSPALTAADVRAMDPADLVSLGGIFAGFLMPKSLKASMESPSS-------- 111
WP_047836209.1_B.gladioli_NGJ1    LCKVLPRITSPALTAADVRAMDPADLVSLGGIFAGFLMPKSLKASMESPSA-------- 111
WP_047836209.1_B.gladioli         LCKVLPRITSPALTAADVRAMDPADLVSLGGIFAGFLMPKSLKASMESPSA-------- 111
WP_013696218.1_B.gladioli_BSR3    LCKVLPRITSPALTAADVRAMDPADLVSLGGIFAGFLMPKSLKASMESPSA-------- 111
WP_126928254.1_S.maltophilia      VATLLPRITEPTLTAADVNKLDPADLVAIGTATAGFFLPKAQQEFLAV----------- 103
WP_071615213.1_Rs_wt              LHTVLPRITTPTLTTADVSKMDPADLTQLASEVSGFLLPKRLKEDSYPSAPTTPSPMLQ 108
WP_197369877.1_R.solanacearum     LHTVLPRITTPTLTTADVSKLDPADLTQLAVEVTGFLLTKAQQQDTFPTESKTPPQTSA 118
                                  : .:***** *:**:***  :*****. :.   :**:: *  :
```

Figure S1. Sequence alignment of Bg_9562 with homologs of different *Burkholderia gladioli* strains and other plant associated bacteria (*Ralstonia solanacearum* and *Stenotrophomonas maltophilia*).

Figure S2

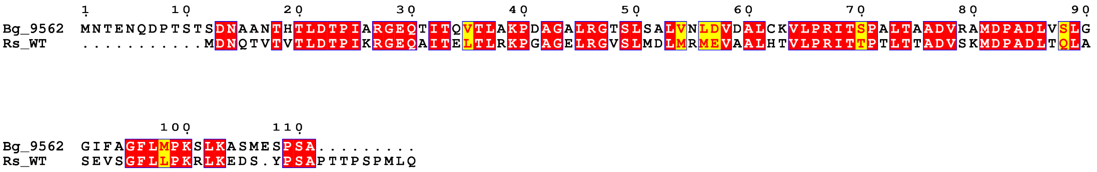

Figure S2. Sequence alignment of Bg_9562 and wild type homolog from *Ralstonia solanacerum.* The residues in red are similar and those enclosed in red box indicate absolute conservation.

Table S1:List of primers used in this study

Primer	Sequence (5’-3’)
Bg_Δ16N_F	CGCGGGATCCAACACGCACACGCTCGACACG
Bg_Δ8C _F	CGATCGGATCCATGAACACGGAAAACCAG
Bg_Δ8C_R	GGGGATTCCATGCTCTATTTCAGCGACTTCG
